# A non-traditional approach to cryopreservation by ultra-rapid cooling for human mesenchymal stem cells

**DOI:** 10.1371/journal.pone.0220055

**Published:** 2019-07-22

**Authors:** Tiziana Irdani, Benedetta Mazzanti, Lara Ballerini, Riccardo Saccardi, Renato Torre

**Affiliations:** 1 CREA Research Centre for Plant Protection and Certification, Florence, Italy; 2 Department of Experimental and Clinical Medicine, Careggi University Hospital, Florence, Italy; 3 Department of Cellular Therapies and Transfusion Careggi, Florence, Italy; 4 European Laboratory for Non-linear Spectroscopy (LENS) and Dipartimento di Fisica e Astronomia, Università degli Studi di Firenze, Sesto Fiorentino, Florence, Italy; National Cancer Institute, UNITED STATES

## Abstract

Cryopreservation is the most common method for long-term cell storage. Successful cryopreservation of cells depends on optimal freezing conditions, freezer storage and a proper thawing technique to minimize the cellular damage that can occur during the cryopreservation process. These factors are especially critical for sensitive stem cells with a consequential and significant impact on viability and functionality. Until now, slow-freezing has been the routine method of cryopreservation but, more recently rapid-cooling techniques have also been proposed. In this study, an ultra-rapid cooling technique [1] was performed for the first time on human mesenchymal stem cells and the effectiveness evaluated in comparison with the conventional slow-freezing procedure. A thin nylon-membrane carrier was used combined with different cryoprotective agents: dimethyl sulfoxide, ethylene glycol and/or trehalose. Various aspects of the low cryoprotective doses and the ultra-rapid cooling procedure of the human mesenchymal stem cells were examined including: the physical properties of the nylon-support, cells encumbrance, viability, proliferation and differentiation. The expression of cell surface markers and apoptosis were also investigated. The study used an ultra-rapid cooling/warming method and showed an overall cell integrity preservation (83–99%), with no significant differences between dimethyl sulfoxide or ethylene glycol treatment (83–87%) and a substantial cell viability of 68% and 51%, respectively. We confirmed a discrepancy also observed by other authors in cell viability and integrity, which implies that caution is necessary when assessing and reporting cell viability data.

## Introduction

Long-term preservation technology using cryopreservation are aimed at preserving the biological properties of cells, tissues, organs and small organisms by decreasing their temperature (to -120°C) so as to halt their metabolism. Such a practice plays a key role in biological sciences and medicine, particularly in transplantation and regenerative specialities. The most frequent clinical application of cryopreservation is for bone marrow transplantation, which is a treatment for many malignant and hereditary diseases of the haematopoietic system. More than 40.000 autologous and allogeneic, including cord blood transplants are performed annually in Europe, half of them using haematopoietic stem cells (HSCs) that have been previously frozen and thawed at the bedside. Cryopreservation of gametes is also widely employed in reproductive medicine, whilst the need for a widespread storage of clinical-grade cellular grafts is envisaged in the emerging field of regenerative medicine. Indeed, the banking of cells and tissues of either healthy donors or patients has been increasingly developed in the past decade to provide scientists of different disciplines with viable bio-samples for basic and applied research, as well as in life sciences.

Mesenchymal stromal cells, known as MSCs are a rare heterogeneous population that can be isolated by plastic adherence from different adult tissues, including bone marrow (BM) and adipose tissue (AT). Due to their differentiative, immunomodulatory and *in vitro* expansive potential these cells have become interesting in the field of cellular therapy [2–4]. MSCs are cryopreserved using slow freezing protocols utilizing dimethyl sulfoxide (DMSO) as the cryoprotectant and thus they retain their biological properties, viability and differentiation potential post-thaw [4,5]. Recent studies have confirmed that 10% DMSO and slow cooling/rapid warming does not affect the viability or differentiation potential of adipose-derived MSCs [5].

Conventional cryopreservation is based on slow freezing technology, where samples are cooled through a slow stepwise procedure in a medium supplemented with a Cryoprotectant (CP). Such technology is commonly used albeit no perfectly standardized for clinical and experimental settings [6], because cells are exposed to many thermal, chemical and mechanical injuries resulting in some degree of cell loss, viability impairment and changes of their biological properties, such as abnormal differentiation of SCs [6]. So far, the quality assessment of freezing has been based on a number of biological indicators of the thawed product, such as viability assay, proliferation potential and evaluation of surface molecules [7]. Optimization of cryopreservation is carried out through the fine tuning of several parameters, including cell concentration or thickness of the tissue to be frozen, cooling rates, sample volumes as well as type, concentration of CPs and the storage temperature. Such parameters are indirectly adjusted by assessing the biological properties of the sample after thawing and, once a procedure is validated for a given product, different types of cells, tissues or small organisms, it can be transferred to the daily practice [1,3]. In the clinical setting, quality controls are usually requested by international standards, by thawing a satellite aliquot before use (www.factwebsite.org/Standards/). So far, the structural and molecular mechanisms of the cryopreservation process are poorly investigated and a quality control of the samples after cryopreservation, based on physical parameters, is still lacking in the daily practice. Vitrification is defined as glass–like solidification of supporting solutions and living cells, completely preventing ice crystal formation during cooling and warming, which is crucial to avoid mechanical injuries to the cells. Vitrification is usually achieved by adding high concentrations of CPs or applying a very fast cooling rate which skips the crystallisation temperature before ice nucleation initiates. In the last decade, vitrification has been reported to be superior to conventional freezing in preserving various cell types and tissues including vascular grafts, human embryos and liver slices.

In particular, the non-equilibrium states of liquid water appear to be relevant to understand the competition between crystal or glass formation [8,9]. In pure water and/or in water mixtures the rate of cooling is a key parameter to avoid the nucleation processes opening the path to glass transition and vitrification phenomena. In particular, the vitrification as opposed to crystallization, is crucial for the preservation of integrity and biological functionality. A series of studies have been devoted to implement innovative cryopreservation techniques based on the increase cooling rates of different biological samples [7,10]. The cooling rate was increased beyond the speed used in earlier studies [7,11] and we showed how this methodology was particularly successful for micro- and small-organisms in different settings [1,12,13]. This approach meant that the concentration of CP could be reduced by increasing the cooling/warming rates up to an ultra-rapid cooling (URC) thus defining a low-CP technique (LCPT) capable of inducing sample vitrification [1]. So far, vitrification has been mostly employed in the experimental setting and has not yet been validated for a large-scale clinical application. The LCPT ensures the preservation of different cell types using the same procedure. It is performed here on human mesenchymal stem cells thus providing a new method applicable to human cell cryostorage.

## Materials and methods

### Mesenchymal stem cell culture and characterization

Human MSCs (hMSCs) were isolated from BM of healthy donor. The use of human bone marrow derived Mesenchymal Stromal cells for research was approved by the Careggi University Hospital Ethic Committee, Florence; Italy Local Ethical Committee (Prot. n. 2007/0026691 dated July 05–2007). Written informed consent was obtained from each bone marrow donor. Whole BM was collected from the iliac crest in tubes containing acid citrate dextrose and centrifuged for 10 minutes at 700g; the interface between plasma and the red cell pellet (buffy-coat) was recovered and total nucleated cells were then plated in 75cm^2^ flasks (1.6x10^5^ cells/cm^2^) in Dulbecco’s modified Eagle’s medium with low glucose, (DMEM-LG; Gibco, Invitrogen, Milan, Italy), and FBS (HyClone, South Logan, Utah) at 10% added and incubated at 37°C in a humidified atmosphere containing 95% air and 5% CO_2_. On reaching confluence, the adherent cells were harvested with 0.05% trypsin and 0.02% EDTA (Eurobio) and re-suspended in a complete medium (primary culture, P0). Cells were plated again at 10^4^ cells/cm^2^ in 100-mm dishes (P1); expansion of the cells was obtained with successive cycles of trypsinization and reseeding. In addition, the ability of MSCs to differentiate along osteogenic and adipogenic lineages was assayed, as previously described [14].

MSC were analysed using flow cytometry for the expression of the following conjugated monoclonal antibodies: CD34-PE, CD45-FITC, CD14-PE (in order to quantify haemapoietic-monocytic contamination); CD29-PE, CD44-FITC, CD166-PE, CD90-PE, CD73-PE, HLA-DP Q R FITC, HLA-ABC FITC (BD Pharmingen) and CD105-PE (Ancell). Flow cytometric analysis was performed on FACSCanto II (Becton Dickinson) as previously described [14,15].

### MSC cryopreservation by SF

Following trypsinization, the harvested hMSCs were gently suspended in a chilled cryopreservation medium (DMEM 20% FBS, 10% DMSO) at 1x10^6^ viable cells/ml. The protocol used in this study based on a slow freezing procedure that employs 10% solution of cold DMSO added immediately before that cells were put at -80°C. Thus, cells were equilibrated in a cold freezing medium (+4°C) for 1 minute and 1 ml was loaded into 2 ml cryovials [16,17,18]. Vials were cryopreserved for one night in nitrogen vapour phase (-80°C), before storage at -150°C.

### MSC cryopreservation by LCPT-URC

Following trypsinization the harvested hMSCs were gently suspended with a concentration of 1x10^6^ cells/ml in cryopreservation medium: DMEM+0.5M trehalose at room temperature (r.t.), for 1 min; DMEM+ DMSO 10% at +4°C, for 1 min; DMEM+ EG 10%, r.t., for 10 min; DMEM+0.5M trehalose at r.t. + DMSO 10% at +4°C, for 1 min; DMEM+0.5M trehalose + EG 10%, at r.t. for 10 min. Cells equilibrated in freezing medium at r.t. were loaded onto strips 20x20 mm in size of nylon membrane (NYC, Roche) as aliquots >50 μl. After loading, the membranes containing cells were left to dry for a few minutes under the laminar hood and then rapidly cooled in liquid nitrogen. The URC rate was considered at least 10^5^ °C/min as estimated in [1]. Membranes free from any extra cryoprotectant volumes were stored in cooled cryovials immersed in liquid nitrogen and then placed in the nitrogen-vapour phase of the tank (approx. -150°C).

### Quantification of residual solution on NYC before cooling

Measurements of the cryoprotective mixture residue in the sample loaded on NYC, during the evaporation process at room temperature were taken. This enabled us to approximately calculate the extra-liquid volume presents on the wet sample, just before the URC process occurred. We load nylon-membranes of about 20x20 mm in size with 50 μl of water-10% EG mixture then measured the weight of the wet membranes at different intervals of time, from 1 to 20 minutes. After 20 minutes, the residual cryoprotectant was about 11.5 mg, corresponding to a volume of about 11.5 μl. In the hypothesis that the residual liquid volume spread uniformly over the whole membrane, approx. 400 mm^2^, we estimated that the thickness of the liquid film would be approx. 28 μm. Since NYC is made by a complex texture of polymeric wires the area under consideration is likely to be greater, hence the calculation of the film thickness was overestimated. However, given the micrometric thickness of the residual film was expected that it easily vitrifies by an ultra-rapid cooling.

### Cell thawing

SF vials were rapidly thawed in a 37°C water bath and the cells recovered using a growth medium (DMEM 10% FBS) dilution followed by centrifugation. Membranes treated by URC were rapidly thawed in a 37°C growth medium placed in a water bath at 40–42°C, cells were then recovered by centrifugation. The thawing rate was approx. 10^6^ °C as estimated in [1]. The resultant pellet was resuspended in a fresh growth medium before being seeded onto plates for monitoring cell adhesion and growth development.

### Cell morphology and proliferation assay

The cell morphology of hMSCs was analysed 3 days after thawing by light microscopy. Pictures were taken at 10X magnification. After thawing, cells were plated at a density of at least 5x10^5^. Cells were grown up to the confluence for 15–20 days, then trypsinized and analysed for viability and phenotype analysis using flow cytometry. The proliferation capacity of MSCs was estimated calculating the ratio between cells number plated and recovered.

### Cell apoptosis analysis using annexin V and 7-Amino-ActinomycinD (7-AAD) staining, pre- and post-cryopreservation

Cells were collected and stained soon after thawing, apoptosis and necrosis have been evaluated by using 7-AAD (BD) and annexin V-FITC Kit (Miltenyi Biotec) following the manufacturer's instructions. Analysis of annexin V/7-AAD-stained cells using flow cytometry allows for quantification of cells that are (i) annexin V-negative and 7-AAD-negative (viable cells), (ii) annexin V-positive and 7-AAD-negative (early apoptotic), (iii) annexin V-positive and 7-AAD -positive (late apoptotic) and (iv) annexin V-negative and 7-AAD -positive (necrotic).

### Statistical analysis

Student’s t-test was used for statistical evaluation. Level of significance was set at p<0.05. All the experiments described were performed in triplicate.

## Results

### Physic-chemical properties of carriers and species influencing cryopreservation

**Fig 1:**
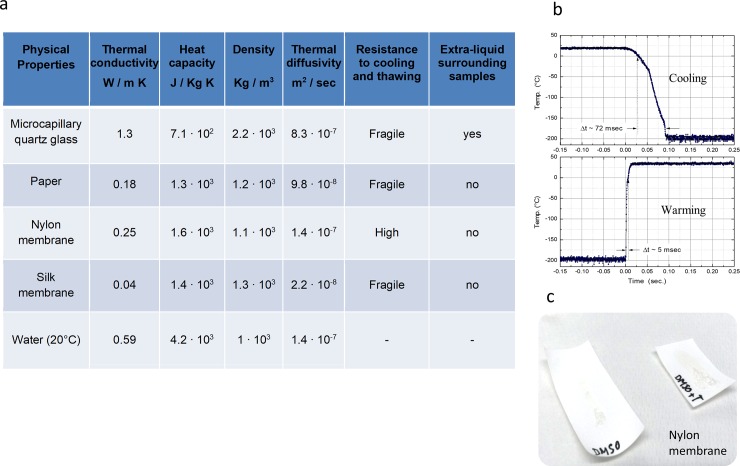
Cryo system supports and thermo-physical properties. In the panel a) the thermo-physical properties of a series of cryo-system supports: thermal conductivity, *k*, heat capacity, *C_p_*, density *ρ* and thermal diffusivity, $\alpha =\frac{k}{C_{p}\rho }$. In the panel b) the measurement of the cooling and heating processes of a thermocouple mimic the cryo-system support, this data has been previously reported see ref. 1. In panel c) the nylon membranes employed as support.

Cell vitrification can be achieved with low CPs doses using open carriers that provide fast cooling and warming processes. The reduction of extra-liquid around the samples and the rapid immersion of the samples in the liquid nitrogen (LN_2_) are important features of innovative cryopreservation protocols. The physical parameters of NYC were relevant in order to implement the rapid cooling of the sample. In Fig 1(A) we report a table with some thermo-physical parameters in comparison to the other most commonly employed carriers.

**Fig 2:**
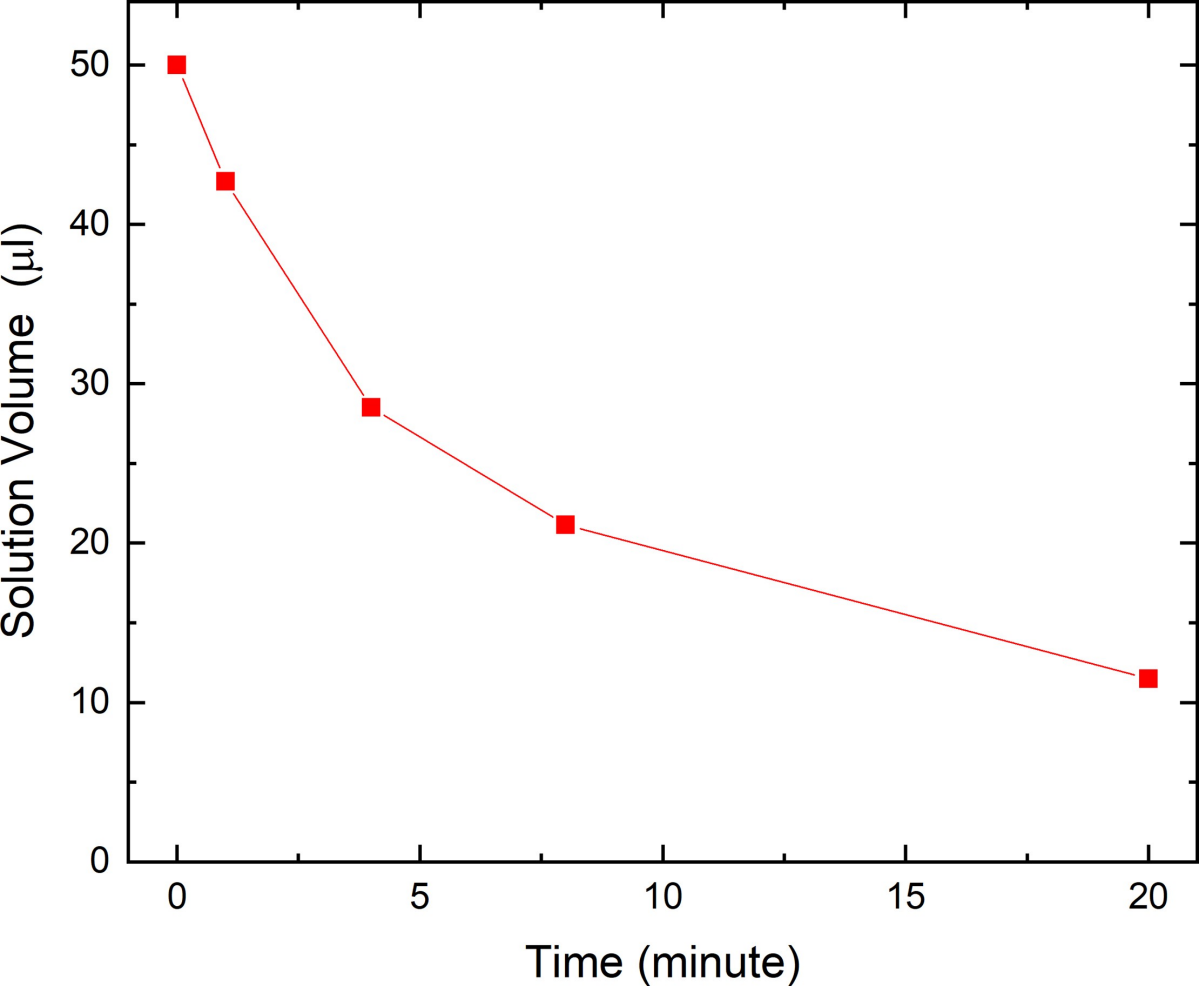
The evaporation trend of the cryoprotective mixture up-loaded onto the NYC.

The URC procedure consists of a direct and deep immersion of the sample in a liquid nitrogen bath. The heat transfer process that takes place when the sample is rapidly immersed into LN_2_ is rather a complex evaluation. The thermal transfer is hindered by the generation of a nitrogen vapour film around the sample that acts as a thermal insulator [19]. This phenomenon is demonstrated by the presence of a high level of asymmetry between cooling and warming rates observed in the temperature measurements performed with a thermocouple, see Fig 1(B). Moreover, this effect produces the surprising result that the samples characterised by a lower thermal conductivities reveal faster cooling rates [20]. This is because the film of nitrogen vapour is reduced and hence a more efficient thermal transfer between LN_2_ and the sample can occur. The data reported in Fig 1(A) show that all the materials have similar thermal diffusivities. Nevertheless, our sample is characterized by a very complex structure that prevents to predict immediately the heat transfer efficiency from the thermo-physical coefficients. Some theoretical models have been developed [7,19] where different thermo-physical parameters were considered in order to describe the cooling behaviour of a body when rapidly immersed into liquid nitrogen; these include the formation of vapour film and some boiling phenomena around the sample/carrier, beside the release of latent heat connected to the eventual phase transitions taking place during the cooling process, as well. Perhaps, a theoretical model for these samples turns to be extremely complex and possibly of further studies. Samples were loaded on NYC in max. 50 μl EG solution (10%) and the drying process was measured and reported, as seen in Fig 2.

In Table 1 other important factors influencing the cryopreservation process such as cell/body size and the water content of the sample, are underlined. The procedure performed in this study assured high survivals of the hMSCs (68%) as well as offering simplicity of up-loading and fast recovery after thawing, in a suitable volume of liquid medium.

**Table 1:** Summary of biological properties and survival percentages by LCPT-URC procedure.

Species	Mean Cell/Body size diameter length (μm)	Mean Volume (μm^3^)	Magnification	Water contents	Survival to LCPT-URC%
**Micro-organisms**	0.5x2	0.65–42	1–65 X	70	**High**^§^
**Mammalian cells**	15–30	10^3^−10^4^	10^3^−10^4^ X	84	**68±20**
**Nematode**	≈68x1.100	4x10^6^ (≈4nl)	10^6^ X	88	**88±3.9**^§^

§ Irdani et al., Cryobiology 2015

### Comparisons among different cryo-techniques and CPs, on hMSCs

In Table 2, we briefly summarize some peculiarities of the most representative cryopreservation procedures from slow freezing to vitrification; details encountered among the conventional (equilibrium) and non-conventional (non-equilibrium) approaches [21]. The more representative parameters consist (1) in the concentration and the equilibrium time of the CPs, since they tend to affect the rate of water transport, nucleation and ice crystal growth. (2) The presence of external liquid, because involves different heat and mass transfer effects. (3) The size of the cell pool loadable. (4) The range of cooling and warming rates and (5) an approximately costs estimation for each procedures; (6) the necessity of special equipment, or simply nitrogen tanks, for maintenance of samples in the cryostorage; (7) the presence or absence of specific carriers. In this study, LCPT-URC appears to preserve survival of hMSCs (51–68%) comparable to those obtained with slow-cooling. Meantime, it allows large amount of cells being loaded on NYC and an easy handling of this support during the thawing and recovery phases. LCPT-URC might be safer during storage and thawing since it avoids prolonged toxic contact with the CPs and the sensible samples.

**Table 2:** Comparison of Ultra Rapid Cooling (LCPT-URC) *versus* Vitrification and Slow-Freezing.

Peculiarities	Vitrification			Slow-freezing
	Equilibrium^§^	Non-Equilibrium^§^		
	Standard	High Cooling	Ultra-Rapid Cooling	Low Freezing rate
**Direct contact in liquid nitrogen**	No	Yes	Yes	No
**Procedure-time**	Slow	Fast	Fast	Slow
**CPs concentration (%)**	High (40–60%)	Low (≥20%)	Low (10%)	Low (10–15%)
**Toxicity risk**	Elevated	Low	Low	Medium
**CPs equilibration-time**	min-hour	min-hour	min	min-hour
**External liquid volume**	≈1 ml	>100 μl	≈10 μl	≈1 ml
**Cell number**	large	small	large	large
**Cooling rate (°C/min)**	Low (1–2)	≈10^3^	≥10^5^	Low (1–2)
**Warming rate (°C/min)**	≈ 10^2^	≈10^3^	≥10^6^	≈18
Estimated costs:				
** Manipulation skill of minimum volumes;**	Good	Good	Good	Good
** Sample loading & recovery**	Easy	Difficult	Easy	Easy
** Special equipment for time-programmed cooling**	No	No	No	Expensive
Materials/carriers: cryo-tube*, straw**, quartz microcapillary**, microdroplets**and nylon membrane***	Cheap*	Expensive**	Less expensive***	Cheap*

Table 2 adapted from ***[1] Irdani et al., 2015

*[5][6]Hunt, 2011; 2017

**[22]Heo et al., 2015; [11] Zhang et al., 2011

^§^ Equilibrium/Non equilibrium concepts were referred as described by Mazur, 1990 [21] and recently by Hunt, 2017 [6].

Cryoprotectants are classified as permeating or non-permeating depending on their ability to traverse the cell membrane [23]. In our experiments two permeating cryoprotectants were employed, DMSO and EG, alone or in combination with trehalose as the only non-permeating ones. The hMSC were briefly treated enzymatically, washed and collected in a tube before proceeding with different treatments.

### LCPT-URC applied to hMSCs: Cell integrity, viability and apoptosis analysis, pre and post-cooling

Cell integrity, viability and apoptosis events were detected before and after the hMSCs thawing, that were treated with one or a combination of three different cryoprotectants. Cells were analysed using 7-AAD and annexin V staining test, since 7-amino-actinomycin D has a high DNA binding constant and so is efficiently excluded by intact cells while annexin V can be used to specifically target and identify apoptotic cells. Results were reported in Table 3 and Table 4, respectively. Cells negative for both, annexin and 7-AAD, were considered alive; cells positive to annexin only were considered as in early apoptosis while, cells positive for both annexin and 7-AAD were considered in late apoptosis, cells positive only to 7-AAD were necrotic. Results showed that cells thawed 2 months after freezing presented mean percentages of cell integrity that was equally high in all the treatments and ranged from 83–99%. Data of cell viability showed more variability in terms of percentages although no significant differences occurred among samples treated by SF with DMSO or by URC with DMSO/EG. Data reported in Tables 3 and 4 showed that viable cells appeared comparable among treatments: 79±3.0%, 68±20% or 51±13%, respectively. Vice versa, significant reductions in viability were observed when URC was applied directly to freshly isolated cells (26±15) %, to trehalose-treated cells (28±2.0) % or to those treated with trehalose together with a second cryoprotective agent, i.e. DMSO (48±11) % or EG (41±16) %. Cells treated with URC showed a higher percentage of early apoptotic cells compared to the SF counterpart: 74±15% for untreated cells; 66±6.0% for trehalose treated cells; 29±15% for DMSO treated cells; 52±10% for DMSO+trehalose treated cells; 44±23% for cells treated with EG and 59±16% for EG+trehalose treated cells. Instead, the percentages of cells in late apoptosis were comparable for all the treatments applied, whether by SF or by URC (Tables 3 and 4).

**Table 3:** Cell integrity and viability analysis was performed on freshly and differently treated hMSC using annexin V (AX) and 7AAD labelling.

MSC	*Cell integrity*	*Viable cells 7AAD-/AX-*	*Early apoptosis 7AAD-/AX+*	*Late apoptosis 7AAD+/AX+*	*Necrotic cells 7AAD+/AX-*	*p-value <0*.*05*
**Freshly isolated**	97%±2.0	90%±4.0	3.9%±3.0	0.8%±0.4	2.6%±2.0	
**Trehalose**	94%±4.5	88%±5.0	8.0%±3.0	1.0%±1.0	3.6%±5.0	No sign.
**DMSO**	97%±2.0	87%±5.0	9.5%±3.0	1.0%±0.0	2.3%±2.0	No sign.
**DMSO & Trehalose**	99%±0.0	92%±1.0	7.0%±2.0	1.0%±0.0	0.2%±0.0	No sign.
**EG**	99%±1.5	91%±2.0	7.0%±0.5	1.0%±1.0	1.5%±2.0	No sign.
**EG & Trehalose**	94%±7.0	88%±7.0	7.0%±0.0	0.0%±0.0	5.0%±6.0	No sign.

Values show the mean percentage of three experiments ±SD.

**Table 4:** Cell integrity and viability analysis was performed immediately after thawing of treated hMSC, by SF or URC, using annexin V (AX) and 7AAD labelling.

	MSC	*Cell integrity*	*Viable cells 7AAD-/AX-*	*Early apoptosis7AAD-/AX+*	*Late apoptosis7AAD+/AX+*	*Necrotic cells7AAD+/AX-*	*p-value <0*.*05*
**Slow Cooling**	**DMSO**	91%±7.0	79%±3.0	10%±8.0	1.0%±1.0	17%±6.0	
**Ultra-Rapid Cooling**	**Freshly isolated**	99%±0.0	26%±15	74%±15	0.0%±0.0	0.2%±0.0	Sign.
	**Trehalose**	99%±0.6	28%±2.0	66%±6.0	0.1%±0.2	0.2%±0.1	Sign.
	**DMSO**	83%±14	68%±20	29%±15	1.0%±1.0	14%±10	No sign.
	**DMSO** & **Trehalose**	99%±0.0	48%±11	52%±10	0.1%±0.1	0.2%±0.2	Sign.
	**EG**	87%±11	51%±13	44%±23	1.0%±0.6	11%±9.5	No sign.
	**EG** & **Trehalose**	99%±0.2	41%±16	59%±16	0.0%±0.0	0.0%±0.0	Sign.

Values show the mean percentage of three experiments ±SD.

**Fig 3:**
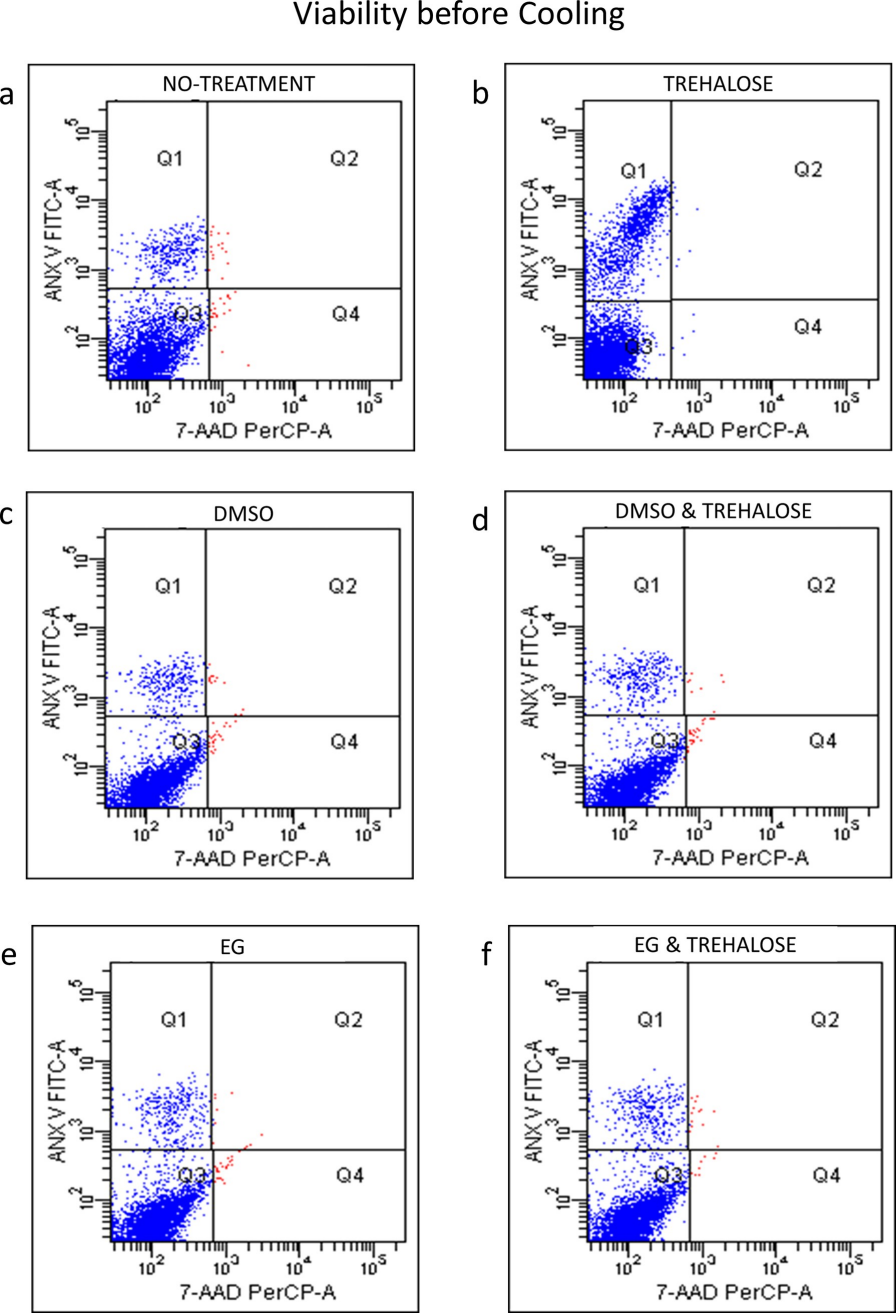
Representative fluorescence activated cell sorting of hMSC for 7-AAD before cooling and after different treatments.

In Fig 3, FACS data show pre-cooling viability of the cells, simply loaded on to a nylon-membrane as freshly isolated cells, or after treatments with one or two different CPs. DMSO, EG and trehalose or a combination of these, appear to maintain highly intact cells with a mean viability of 97%. The NYC proves to be a useful support for hMSCs as well, allowing a fast recovery just after a rapid immersion in a liquid medium.

**Fig 4:**
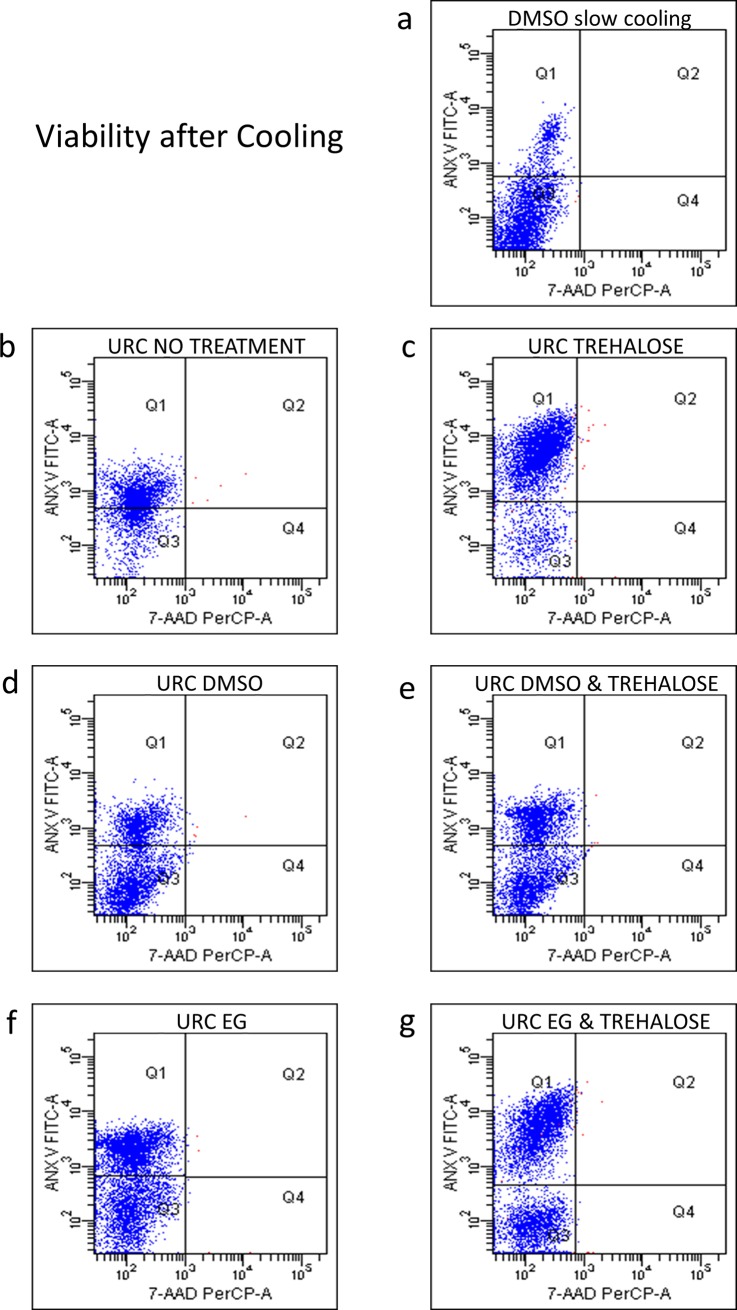
Cytometric analysis of hMSC performed using 7-AAD and annexin after cooling. The images show the viability of cells after 1 month in storage and treated by SF (a) DMSO or by URC procedure of: (b) un-treated cells, (c) trehalose, (d) DMSO, (e) DMSO and trehalose, (f) EG, (g) EG and trehalose treated cells.

Successively, in Fig 4 the FACS post-cooling data show the viability of hMSCs after immersion in LN_2_ bath and storage in nitrogen vapour phase. All treatments applied showed very high recoveries in term of cell integrity, with no significative differences between the average of 94% for URC treated cells compared to that observed for SF treatment of 91%.

In Fig 4 the typical two-channel (FITC-annexin and 7-AAD) flow cytometry data of a representative experiment of hMSCs cryopreservation by URC vitrification, with quadrant gates showing four populations. The corresponding quantitative data from at least three independent experiments are summarized in Table 4.

### Post-thaw hMSC immunophenotype, growth and proliferation

**Fig 5:**
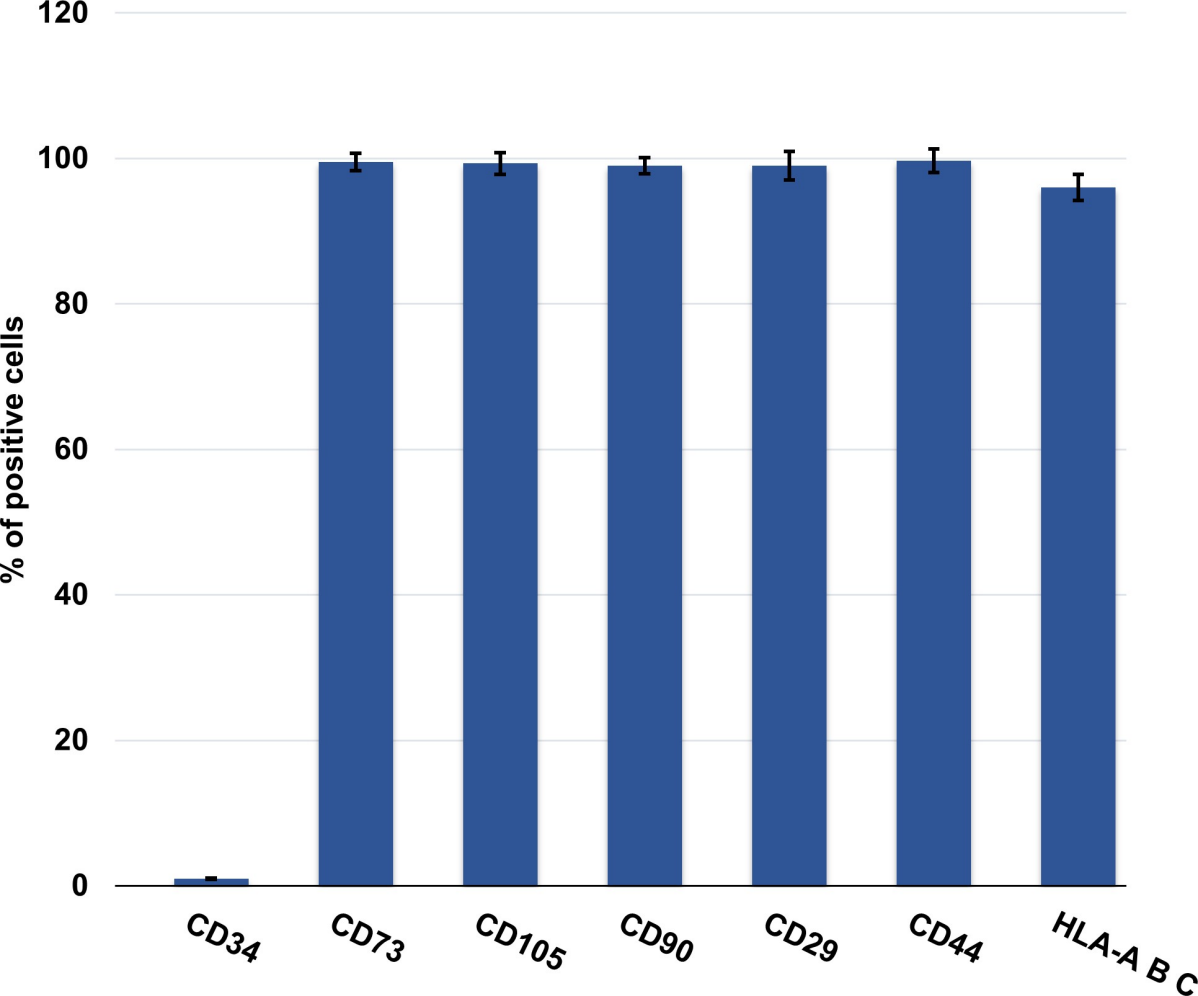
Expression level of positive and negative mesenchymal-associated markers.

Both SF and URC thawed hMSCs resulted positive for the classical mesenchymal markers CD90, CD73, CD105, CD29, CD44 and HLA ABC and negative for the CD34 haematopoietic marker as shown in Fig 5, thus suggesting that the cryopreservation method does not influence the hMSC immunophenotype.

**Fig 6:**
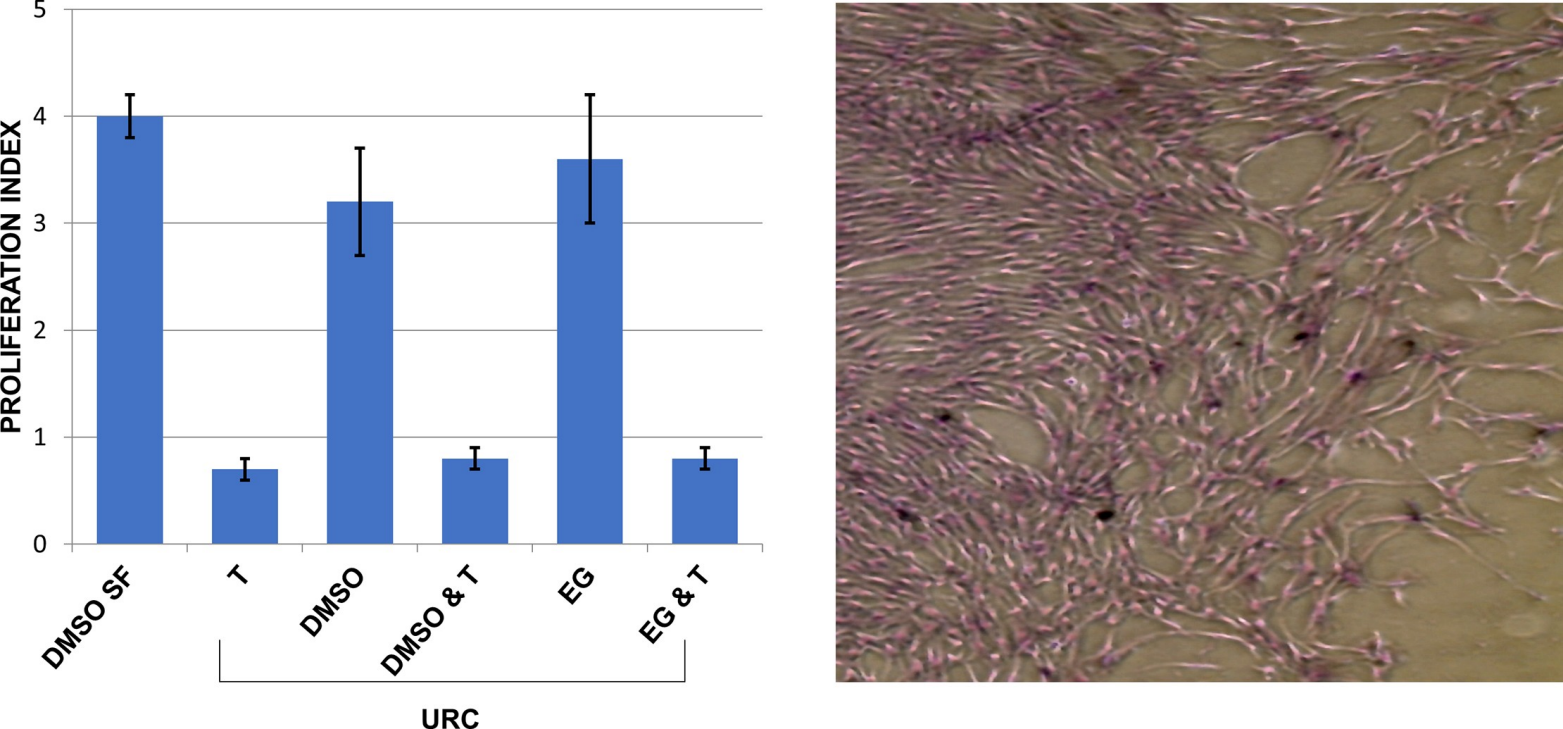
Proliferation index and cell growth after thawing.

**Fig 7:**
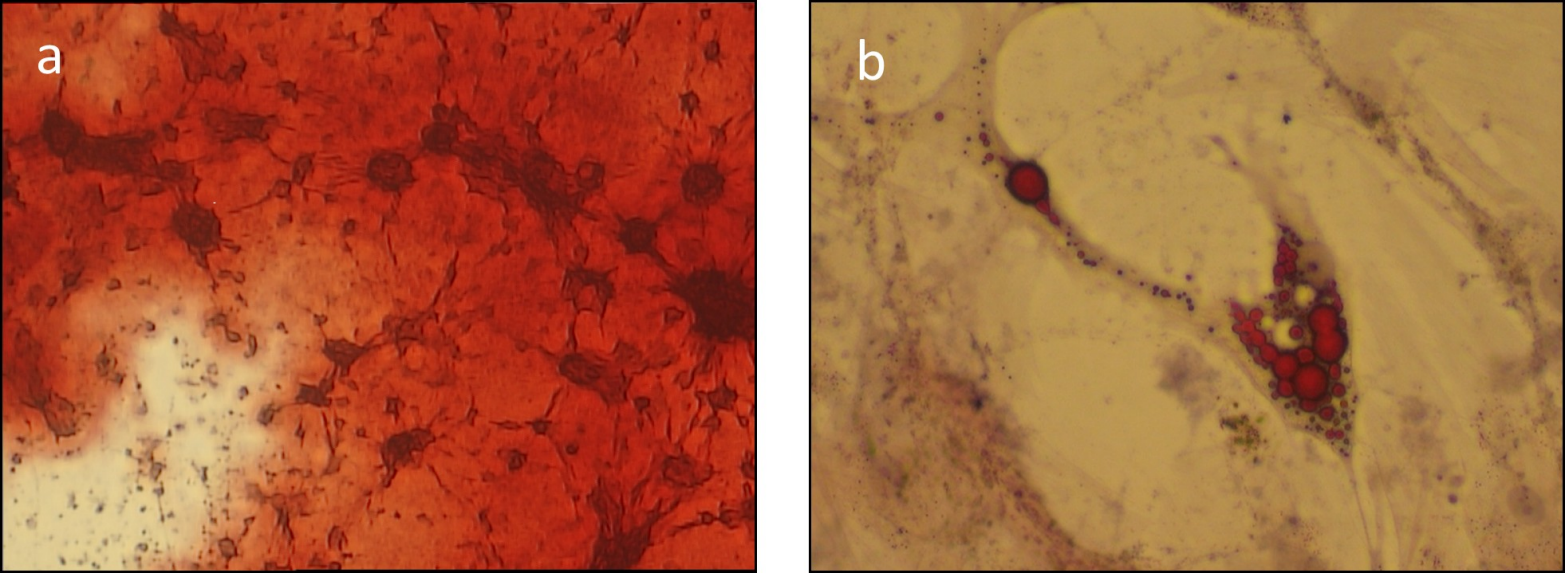
Osteogenesis- and adipogenesis- induced differentiation; (a) Osteogenic differentiation: Alizarin red staining of the hMSCs following 3 weeks induction in osteogenic differentiation medium. (b) Adipogenic differentiation: Oil red O staining of the fat vacuoles in hMSCs following 3 weeks of induction in adipogenic differentiation medium.

Attachment efficacy was assessed after thawing differently treated hMSCs. The percentage of adherent cells was measured by the ratio of the number of hMSC seeded immediately after thawing and those recovered by culture trypsinization, in a humidified atmosphere of 95% air and 5% CO_2_. The attachment capability (% of adherent cells) was comparable to those of viable cells (non-apoptotic cells) detected by FACS for both the procedures used: SF and URC as shown in Table 4. Proliferation of thawed cells was evaluated at the third passage of culture plating. Soon after thawing, cells appear to proliferate very slowly compared to fresh ones to reach at confluence in 15–20 days. A delay in cell proliferation is commonly linked to the concentration of CPs (DMSO, EG) and this delay became even longer when trehalose (0.5M) was added. However, at the third passage the proliferation index of thawed hMSCs was relatively high and comparable either in cells treated with DMSO or EG, by SF or URC procedures. Vice versa, proliferation index of trehalose treated cells were negatively affected as reported in Fig 6. Remarkably, URC procedure applied to hMSC treated with the different cryoprotectants still maintained the capability of cells to differentiate toward osteogenic and adipogenic lineage after thawing (Fig 7).

## Discussion

Vitrification phenomena are the direct transformation of aqueous solutions into glass phases, after direct exposition to liquid nitrogen. Two approaches have been experimented so far to preserve cells based on equilibrium or nonequilibrium vitrification. The first approach employs multi-molar CPs mixtures in stepwise exposition, before immersion in liquid nitrogen. The equilibrium freezing defined by Mazur, 1990 [21] consists in the capacity of cooled cells to maintain the chemical potential of their intracellular water close to that of extracellular solution when water partially freezes. This effect occurs when cells are cooled slowly, e.g. approximately 1°C/min. to about -70°C. The alternative, is a nonequilibrium vitrification that adopts rapid or ultra-rapid cooling rates in addition to mixtures with lower doses of CPs; these cooling rates exclude the re-equilibration between intra- and extra-cellular water by efflux processes, but the equilibrium can be restored by intracellular ice formation. At ultra-rapid cooling rate (10^4^−10^6^°C/min) the biological system can vitrify by forming a glass state thus excluding ice crystals and damages, as well. Here, a new effective cryopreservation technique for hMSCs has been established. It is based on a low cryoprotectant technique and ultra-rapid cooling that had already been applied to several other cells and species [1] and here, for the first time, performed with hMSCs. A non-conventional ultra-rapid cooling by LCPT and free of DMSO provide us with further information about hMSCs behaviour in response to the cryopreservation process compared to the standard slow-cooling method (SF) commonly used. The LCPT-URC prevents ice-crystals assuring cell vitrification as already demonstrated [1], even after a short incubation in cryoprotectant/s because a thin NYC was employed as a carrier. It is well known that the cryopreservation process is influenced by several factors: freshness and cell concentration, CPs types and doses, sample volumes and cooling/thawing execution and all these factors play important roles in viability decline of the cryopreserved cells/samples, after thawing. The role of water in all these processes is particularly relevant and it remains puzzling. Indeed, our previous study already demonstrated that vitrification can occur, even in multicellular organism such as nematodes, using the LCPT-URC technique that is based on low-cryoprotective doses and ultra-rapid cooling [1]. Meanwhile, the LCPT-URC also allowed a rapid re-warming thus preventing the ice recrystallization during thawing that can lead to further cell mortality. It is well known that preservation of biological activities is intrinsically connected with several physical-chemical parameters and, the LCPT-URC, employing NYC as a carrier, was shown to be a suitable approach to reduce the sample encumbrance and thus increasing the cooling rate. The attractiveness of NYC lies in its hydrophilicity as well as some other technical advantages such as resistance, sample portability and capacity to limit excess in extra liquid volumes providing a low thermal-conductivity. In fact, the lower the thermal conductivity of the carrier the faster the sample cooling rate will be, as a direct consequence of the reduced effect of nitrogen vaporization around the sample. At this regard, paper and silk membranes should be more indicated exerting a rapid cooling rate due to their lower thermal conductivities. Instead, other disadvantages occurred for both, i.e. excess of fibres release during thawing phase or, an extreme fragility combined to certain hydrophobicity, during cooling or loading, respectively. Moreover, NYC can upload many cells and, in reverse, it offers an easy and fast recovery of the sample during thawing. Furthermore, viability data presented earlier vary substantially depending on the assay employed, as demonstrated and reported by other authors [24]. The method of trypan blue exclusion is generally used, but its sensitivity was shown to produce some ambiguous yields [24]. Thus, in our study the 7-AAD labelling test was employed to check cells integrity while, for the detection of cells viability the more sensitive annexin V/7-AAD assay was adopted, quantitatively assessed by cytofluorimetric analysis. The annexin V/7-AAD assay is a more reliable test in terms of viability since it shown a precise discrimination between cells in both early and late apoptotic states: as well as the necrotic state. Early apoptosis is predominant after the URC approach, compared to necrosis or late apoptotic events. In this study, we demonstrated an overall high cell integrity after LCPT-URC application (83–99%), with no significant differences between the CPs employed, DMSO or EG. However, a consistent cell viability was also obtained with treatments of DMSO or EG (68% and 51% respectively). Vitality of thawed hMSCs were also evaluated after just adding trehalose and in combination with either DMSO or EG. A severe reduction in viability was observed with trehalose treated cells, where survivals dropped up to 28% with a substantial increase in early-apoptotic ones (66%). Again, similar results were achieved when a combination of trehalose and DMSO or EG, was applied.

Interestingly, although further investigations are necessary, some good results in cell viability were obtained without any treatment of cells before cooling (up to 41%). The optimization of this process may be an important challenge to solve some of the problems in clinical application of hMSCs. Importantly, it remains to be further investigated on the precise increment in early apoptotic fractions following the URC-treatment; although other authors have suggested that a prolonged period post thawing, at least 24 hrs, might allow these processes to be reversed thereby avoiding apoptosis [21]. Notably, our results underline the discrepancy already observed elsewhere [25] between the rate of cell viability and integrity after thawing (Table 4), revealing the importance of a substantial distinction in the effective number of living cells, after cryopreservation. Successively, we further demonstrated that thawed cells dealt with non-conventional LCPT-URC had no impact on the immunophenotype of hMSCs that maintained the full expression of the typical mesenchymal molecular markers, CD73, CD105, CD90, CD29, CD44, HLA-ABC (positive) and CD34 negative. Although immediately after thawing the cells appear physiologically slower and slightly stunned, they later resume their natural proliferative capacity and even their capacity towards osteogenic and adipogenic differentiation.

## Conclusions

In summary, the LCPT-URC of hMSCs was demonstrated as a possible alternative for cell cryostorage, as it maintains the cell viability, growth kinetics and the multilineage differentiation, upon thawing. Further tests are necessary to demonstrate the increase of early apoptotic cells, perhaps adopting longer intervals of time between thawing and the vitality test. Nevertheless, LCPT-URC not only allowed the use of a small amount of DMSO/EG, but it also goes in the direction of complete removal of any external CPs, before the cell storage. This can limit the side effects such as toxicity from cryoprotectants, which were frequently observed at the bedside and even the possibilities of a complete absence of cryoprotective usage, perhaps eliminating any adverse effects thus enhancing the quality of therapeutic care. These results offer interesting possibilities for studies of other biological materials, such as cord-blood cells and tissues, with innovative perspectives for scientific and medical applications.

## Abbreviations

hMSCs: human mesenchymal stem cells
SCs: stem cells
NYC: nylon carrier
DMSO: dimethyl sulfoxide
EG: Ethylene glycol
URC: ultra-rapid cooling
LCPT: low cryoprotectant technique
